# Lung Transplantation Outcomes and Peritransplant Sirolimus Use in Lymphangioleiomyomatosis

**DOI:** 10.1016/j.atssr.2024.07.011

**Published:** 2024-07-26

**Authors:** Emily L. Larson, Reed T. Jenkins, Jessica M. Ruck, Laura B. Zeiser, Alice L. Zhou, Alfred J. Casillan, Dorry L. Segev, Allan B. Massie, Jinny S. Ha, Pali D. Shah, Christian A. Merlo, Errol L. Bush

**Affiliations:** 1 Division of Thoracic Surgery, Department of Surgery, Johns Hopkins Hospital, Baltimore, Maryland; 2 Department of Surgery, NYU Langone School of Medicine, New York, New York; 3 Department of Population Health, NYU Grossman School of Medicine and Langone Health, New York, New York; 4 Scientific Registry of Transplant Recipients, Minneapolis, Minnesota; 5 Pulmonary and Critical Care Medicine, Department of Medicine, Johns Hopkins Hospital, Baltimore, Maryland

## Abstract

**Background:**

With the introduction of sirolimus as medical therapy for lymphangioleiomyomatosis (LAM), an updated evaluation of LAM lung transplant (LT) outcomes and characterization of peritransplant sirolimus use is needed.

**Methods:**

We identified adult LT recipients from 2005-2021 using the Scientific Registry of Transplant Recipients database and stratified by diagnosis (LAM vs other). Multivariable Cox regression was performed to calculate the adjusted hazard ratio for LAM vs other diagnoses. A pharmacy claims database was linked to provide sirolimus prescription information, and a subgroup analysis comparing outcomes with pre- vs posttransplant sirolimus use was performed.

**Results:**

Of 32,337 recipients identified, 156 (0.5%) were diagnosed with LAM. Operative complications, including airway dehiscence, did not significantly differ between groups. After adjusting for donor and recipient characteristics, LAM diagnosis was associated with 45% lower mortality than other diagnoses. Among recipients with pharmacy data, 32% were prescribed sirolimus at any point. Compared with only post-LT use only, recipients with pre-LT sirolimus use had increased mortality (log-rank *P* = .003).

**Conclusions:**

This study supports lung transplant as a treatment for severe pulmonary LAM and identifies increased mortality associated with pre-LT sirolimus, though this may be due to uncharacterized baseline differences.

**In Short**

▪ Lung transplant remains effective for end-stage lymphangioleiomyomatosis, with improved survival compared to other indications.
▪ Initiation of sirolimus pretransplant in lymphangioleiomyomatosis patients was associated with increased mortality, though possibly is due to unreported factors.

Lymphangioleiomyomatosis (LAM) is a rare, progressive disease that can result in cystic lung destruction. The pathogenesis of LAM is overactivation of the mammalian target of rapamycin complex 1 (mTORC1) pathway.^1^ Sirolimus (rapamycin) is an mTORC1 inhibitor that has been shown to effectively stabilize lung function in LAM after a pivotal 2011 trial and is now considered a first-line agent for patients with moderate to severe pulmonary LAM symptoms.^2^

Patients with severe LAM benefit from lung transplant (LT).^3^^,^^4^ One of the chief concerns of sirolimus use is its association with increased rates of airway dehiscence of the bronchial anastomosis post-LT.^5^ Optimal sirolimus use in the peri-LT period has not been established.^6^^,^^7^ Specifically, though sirolimus has been shown to benefit patients with LAM, the impact of initiating sirolimus before vs after LT is unknown.

Therefore, we sought to evaluate peri-LT sirolimus use in patients with LAM, as well as provide updated assessment of outcomes of LT in patients with LAM.

## Material and Methods

### Data Source

This study used data from the Scientific Registry of Transplant Recipients (SRTR). The SRTR data system includes data on all donor, waitlisted candidates, and transplant recipients in the United States, submitted by the members of the Organ Procurement and Transplantation Network. The Health Resources and Services Administration, US Department of Health and Human Services, provides oversight to the activities of the Organ Procurement and Transplantation Network and SRTR contractors. In SRTR, mortality is reported by individual transplant centers, and ascertainment is supplemented through linkage to the Social Security Master Death File. This data set has previously been described.^8^

The SRTR database was merged with Symphony Health’s pharmacy claims database for sirolimus use data. This pharmacy database includes point-of-sale prescription data, non-retail invoice data, and demographic data from commercial and Managed Medicaid and Medicare Advantage pharmacy claims data of over 280 million patients across the United States and approximately 65% of the US specialty market.^9^

### Study Population

Adult (≥ 18 years) recipients undergoing lung-only transplant between January 2005 and May 2021 were included. Recipients with a prior LT were excluded. Recipients were grouped by diagnosis (LAM vs other).

This study was deemed exempt from review by the Johns Hopkins Medicine institutional review board (NA00042871).

### Donor and Recipient Characteristics

Donor and recipient characteristics were compared for LT recipients with and without LAM and their primary diagnosis using a χ^2^ and Fisher’s exact test for categorical variables and Wilcoxon rank-sum test for continuous variables. Median (interquartile range) is reported for continuous variables, and number (percent) is reported for categorical variables.

### Posttransplant Outcomes

The incidence of post-transplant complications was compared as above. For mortality, time-to-event analysis was performed and visualized using Kaplan-Meier curves. We used multivariable Cox regression to compare risk of posttransplant mortality between recipients with and without LAM, adjusting for baseline characteristics with *P* < .2 on univariate analysis. This provided the adjusted hazard ratio and 95% CI for mortality by diagnosis (LAM vs other). We followed recipients until the outcome of interest or administrative censorship on May 30, 2021.

### Subgroup Analysis of Recipients with Sirolimus Use

We aimed to characterize sirolimus use in patients with LAM and evaluate the impact of starting sirolimus before vs after LT. Given the known benefits of sirolimus use in recipients with LAM, only recipients with a sirolimus prescription were included in this subgroup analysis. Prescription dates were used to characterize sirolimus use as pre- or posttransplant. Recipients with both pre- and posttransplant use were included in the pretransplant group (sirolimus use began pretransplant). Analysis as above was performed to compare recipients with pre- vs posttransplant sirolimus use.

All statistical analyses were performed using R statistical software version 3.6.2 (R Foundation for Statistical Computing) within RStudio statistical software version 1.2.5033 (RStudio).

## Results

### Study Population

Of the 32,337 lung transplant recipients from 2005-2021, 156 received LT for LAM (Table 1). Donors to LAM recipients were more likely to be female and have died of cerebrovascular events. Recipients with a diagnosis of LAM were exclusively female, younger, and had lower body mass index than those with other diagnoses. Recipients with LAM also had lower serum creatinine, spent longer on the waitlist, and were more likely to receive bilateral lung transplant.

**Table 1:** Baseline Characteristics of Patients Undergoing Lung Transplant for Lymphangioleiomyomatosis vs Other Diagnoses in the United States, 2005-2021

Variable	LAM Diagnosis (n = 156)	Other Diagnosis (n = 32,181)	*P* Value
Donor			
Sex (female)	82 (52.6)	12,789 (39.7)	.001
Age, y	34.5 (24.5-48.2)	32 (22-46)	.12
Cause of death			<.001
Anoxia	32 (20.5)	7284 (22.6)	
Cerebrovascular/stroke	72 (46.2)	9939 (30.9)	
CNS tumor	3 (1.9)	206 (0.6)	
Head trauma	45 (28.8)	13,988 (43.5)	
Other	4 (2.6)	764 (2.4)	
Cigarette use (>20 pack-years)	14 (9)	2918 (9.1)	.99
Recipient			
Race			.10
Black	13 (8.3)	2884 (9)	
White	119 (76.3)	26,029 (80.9)	
Other	24 (15.4)	3268 (10.2)	
Sex (female)	156 (100)	13,091 (40.7)	<.001
Age, y	46 (39-54.5)	59 (49-64)	<.001
BMI, kg/m^2^	22.6 (19.8-27.7)	25.3 (21.5-28.7)	<.001
Serum creatinine, mg/dL	0.7 (0.6-0.8)	0.8 (0.7-1)	<.001
Total bilirubin, mg/dL	0.5 (0.3-0.6)	0.5 (0.3-0.7)	.41
Time on waitlist, y	0.4 (0.1-1.1)	0.2 (0-0.5)	<.001
Transplant type			<.001
Bilateral	132 (84.6)	22,726 (70.6)	
Single	24 (15.4)	9455 (29.4)	
Ischemic time, h	4.9 (4.1-6.1)	5.1 (4.1-6.2)	.23

Continuous variables reported as median (interquartile range), and categorical variables reported as n (%).

BMI, body mass index; CNS, central nervous system; LAM, lymphangioleiomyomatosis.

### Posttransplant Outcomes

**Figure 1:**
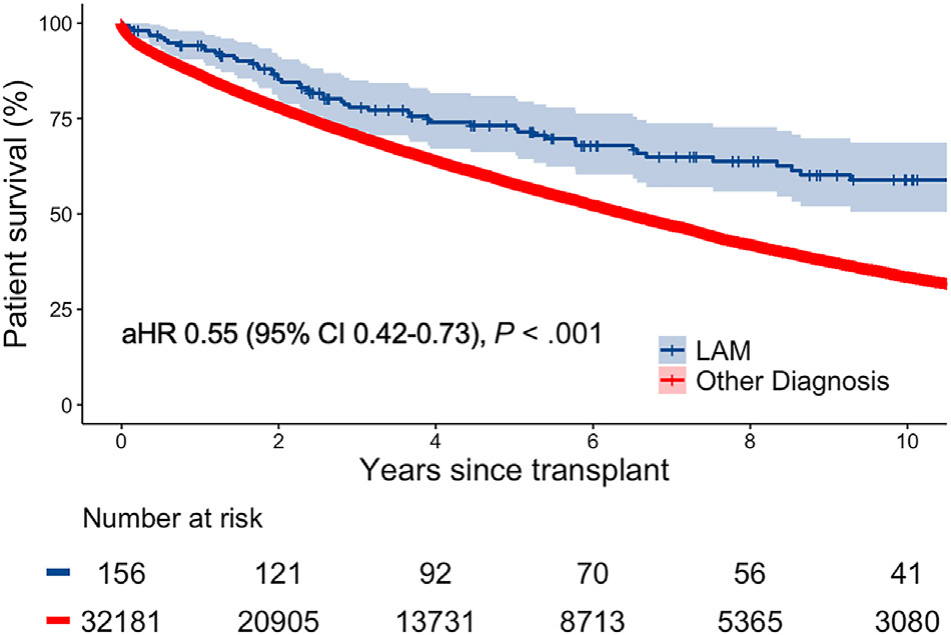
Post-lung transplant survival and 95% CI for recipients with lymphangioleiomyomatosis (LAM) vs other diagnoses in the United States, 2005-2021. (aHR, adjusted hazard ratio.)

Post-transplant complications were similar between LAM and non-LAM recipients (Table 2). However, LAM recipients had lower mortality than non-LAM recipients (log rank *P* < .001), with median (95% CI) survival for LAM recipients of 14.3 (lower 95% CI, 11.0) years and for non-LAM recipients of 6.4 (6.3-6.6) years (Figure 1). Even after accounting for donor, recipient, and transplant characteristics, LAM recipients had a 45% lower risk of mortality compared with non-LAM recipients (adjusted hazard ratio, 0.55; 95% CI, 0.42-0.73], *P* < .001). Supplemental Table 1 shows other factors associated with mortality. Within the LAM population, stratification by age (greater or less than 50 years) did not show a difference in mortality (Supplemental Figure).

**Table 2:** Posttransplant Outcomes of Patients Undergoing Lung Transplant for Lymphangioleiomyomatosis vs Other Diagnoses in the United States, 2005-2021

Variable	LAM Diagnosis (n = 156)	Other Diagnosis (n = 32,181)	*P* Value
Median survival, y	14.3 (lower 95% CI 11.0)	6.4 (6.3-6.6)	<.001^a^
Length of stay, d	19 (12-30.5)	17 (12-28)	.11
Acute rejection	10 (6.4)	2305 (7.2)	.84
Airway dehiscence	1 (0.6)	484 (1.5)	.73
Dialysis	6 (3.8)	2278 (7.1)	.18
Reintubation	32 (20.5)	5789 (18)	.48

Survival reported as median (95% CI), continuous variables reported as median (interquartile range), and categorical variables reported as n (%).

LAM, lymphangioleiomyomatosis.

a Log-rank.

### Subgroup Analysis of Recipients with Sirolimus Use

Of the 156 LT recipients with LAM, 101 had pharmacy data. Recipients with pharmacy data were older (median [interquartile range] 49 [41-57] vs 44 [34-50] years, *P* < .001) and had higher body mass index (23.5 [20.4-28.7] vs 21.4 [18.4-24] kg/m^2^, *P* < .001) but otherwise similar to recipients without pharmacy data.

Among recipients with pharmacy data, 32 (32%) were prescribed sirolimus at any point (Supplemental Table 2), of whom 13 (41%) received any sirolimus pre-LT and 23 (72%) received any sirolimus post-LT. Recipients using sirolimus post-LT began a median (IQR) 2.2 (1.0-5.3) years post-LT.

**Figure 2:**
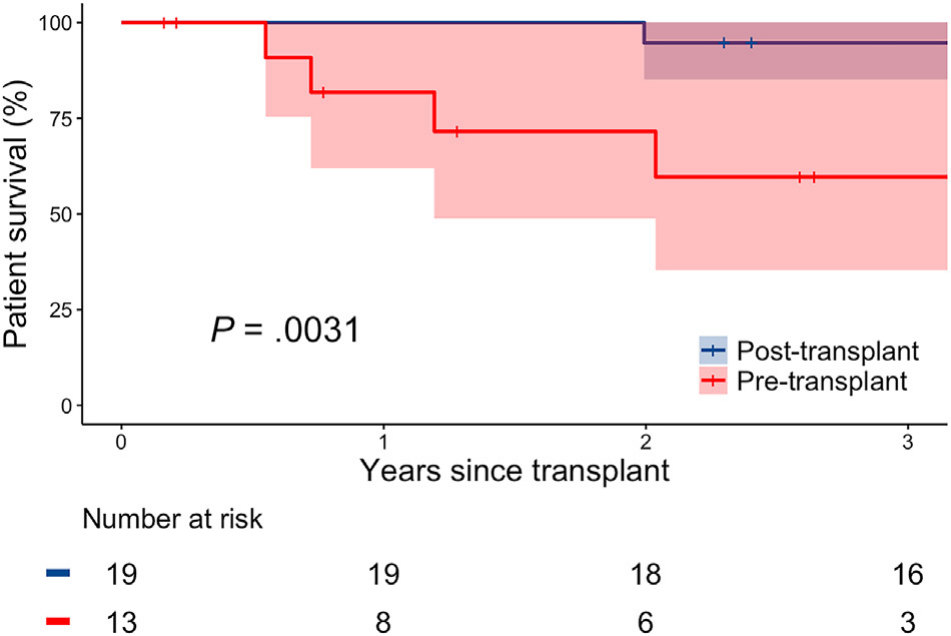
Post-lung transplant survival and 95% CI for recipients undergoing lung transplant for lymphangioleiomyomatosis with pre- vs posttransplant sirolimus use.

Besides older recipient age in the pre-LT group, baseline characteristics for pre- and post-LT sirolimus use recipients were similar (Supplemental Table 3). Post-LT complications were similar between recipients with pre- and post-LT sirolimus use (Supplemental Table 4). Recipients with pre-LT sirolimus use had higher mortality than recipients with only post-LT sirolimus use (Figure 2, median survival 3.7 [lower 95% CI 1.9] years vs beyond 11.8 [lower 95% CI 11.8] years, log-rank *P* = .003).

## Comment

In this national study of lung transplantation and perioperative sirolimus use, we found that patients receiving LT for LAM continued to have significantly longer post-LT survival than other indications. However, among LAM recipients with sirolimus use, pre-LT sirolimus use was associated with increased mortality after LT. Our study is the first to directly examine sirolimus use in the LAM LT population and describe the association of pre- vs posttransplant sirolimus use with LT outcomes.

Two large studies on US cohorts in 2005 and 2019 have shown that patients who receive transplants for LAM have significantly improved survival compared with non-LAM LT recipients.^3^^,^^4^ Most recently, Khawar and colleagues^3^ analyzed a cohort of 24,540 patients who underwent lung transplantation from 2003 to 2017, including 134 with LAM. In this cohort, LAM patients had a median post-LT survival of 12 years. This is in agreement with the 14.3 year median (vs 6.4 years for other indications) post-LT survival for LAM patients observed here.

The markedly superior survival after LT for has been postulated to occur because of the younger age of LAM transplant recipients.^3^ In our cohort, recipients with LAM also had significantly lower body mass index, serum creatinine, and total bilirubin than other LT recipients, supporting LAM patients having improved overall health. This likely only partially explains the observed difference, given that it persisted even after adjustment.

Among those who received sirolimus, pre-LT sirolimus initiation was associated with increased mortality. The half-life of sirolimus is around 62 hours, but optimal peritransplant sirolimus administration remains unclear.^10^ The antiproliferative mechanism of sirolimus yields concern for bronchial anastomosis dehiscence.^5^ However, in our sirolimus cohort, only 1 patient experienced airway dehiscence, and she only received sirolimus post-LT. Therefore, the mortality differences observed between recipients with pre-LT vs post-LT-only sirolimus use might instead be explained by more severe LAM disease or different disease manifestations.

### Limitations

Although our use of national registry data provided the largest LAM cohort to date, data granularity, especially the ability to evaluate extrapulmonary manifestations of LAM, is limited. Additionally, though no significant differences in populations were identified, pharmacy data were only available for 65% of LAM recipients, possibly introducing selection bias. Additionally, indications may vary for posttransplant sirolimus use, as some patients may receive sirolimus due to intolerance of traditional calcineurin inhibitors rather than for LAM. Finally, not all patients filling sirolimus prescriptions may be taking it.

### Conclusions

The findings of this study support lung transplant for end-stage LAM. Among LAM recipients with sirolimus use, initiating sirolimus pre-LT was associated with increased mortality but not bronchial dehiscence. The mortality difference observed is likely attributable to factors unavailable in the national registry data, warranting further exploration to improve post-LT outcomes and waitlist care for patients with LAM.
