# Supplementary material for: Lung Transplantation Outcomes and Peritransplant Sirolimus Use in Lymphangioleiomyomatosis
Source: Ann Thorac Surg Short Rep. 2024 Jul 26;3(1):133–7. doi: 10.1016/j.atssr.2024.07.011 (PMC11910819; doi:10.1016/j.atssr.2024.07.011)
Supplement: Supplementary Figure 1 and Supplementary Tables 1-4 [file mmc1.docx]

**Supplementary Table 1. Multivariate Cox regression for post-transplant survival in lung transplant recipients 2005-2021.**

| **Variable** | **Adjusted Hazard Ratio** | **95% Confidence Interval** | **P** |
| --- | --- | --- | --- |
| Diagnosis |  |  |  |
| Other | Reference | Reference | Reference |
| LAM | 0.55 | 0.42-0.73 | <0.001 |
| Donor age | 1.003 | 1.001-1.004 | <0.001 |
| Donor cause of death |  |  |  |
| Anoxia | Reference | Reference | Reference |
| Cerebrovascular/  stroke | 1.13 | 1.07-1.19 | <0.001 |
| CNS tumor | 1.03 | 0.83-1.27 | 0.81 |
| Head trauma | 1.06 | 1.01-1.11 | 0.02 |
| Other | 1.03 | 0.92-1.16 | 0.62 |
| Cigarette use (>20 pack-years) | 1.12 | 1.06-1.19 | <0.001 |
| Race |  |  |  |
| White | Reference | Reference | Reference |
| Black | 1.04 | 0.98-1.1 | 0.25 |
| Other | 0.92 | 0.86-0.97 | 0.005 |
| Recipient gender |  |  |  |
| Female | Reference | Reference | Reference |
| Male | 1.03 | 1.00-1.07 | 0.09 |
| Recipient age | 1.005 | 1.003-1.006 | <0.001 |
| Recipient creatinine | 1.10 | 1.07-1.13 | <0.001 |
| Recipient total bilirubin | 1.05 | 1.04-1.06 | <0.001 |
| Time on waitlist | 0.97 | 0.96-0.99 | 0.004 |
| Transplant type |  |  |  |
| Bilateral | Reference | Reference | Reference |
| Single | 1.45 | 1.40-1.51 | <0.001 |
| Ischemic time | 1.02 | 1.01-1.03 | <0.001 |

LAM=lymphangioleiomyomatosis, CNS=central nervous system. Variables with a p<0.2 on univariate analysis included in the final multivariate model.

**Supplementary Table 2. Sirolimus use for patients with lymphangioleiomyomatosis (LAM) undergoing lung transplant.**

| **Variable** | **LAM Patients with Sirolimus Use (n=32)** |
| --- | --- |
| Total sirolimus use (years, median [IQR]) | 1.4 (0.6-6.8) |
| Pre-transplant sirolimus use | |
| No preoperative rx | 19 (59%) |
| Last rx filled while on waitlist | 3 (9%) |
| Last rx filled before waitlist | 10 (31%) |
| Post-transplant sirolimus use | |
| No postoperative rx | 9 (28%) |
| First rx filled within 30 days of transplant | 1 (3%) |
| First rx filled after 30 days of transplant | 22 (69%) |

Rx=prescription. Of 101 LAM recipients with pharmacy data available, the 32 with sirolimus prescriptions are characterized above. Continuous variables reported as median (interquartile range), and categorical variables reported as n (%).

**Supplementary Table 3. Baseline characteristics of patients undergoing lung transplant for lymphangioleiomyomatosis (LAM) with pre- vs. post-transplant sirolimus use.**

| **Variable** | **Pre-transplant sirolimus (n=13)** | **Post-transplant only sirolimus (n=19)** | **P** |
| --- | --- | --- | --- |
| **Donor** |  |  |  |
| Gender (female) | 9 (69.2%) | 9 (47.4%) | 0.39 |
| Age | 37 (25-42) | 44 (27-51.5) | 0.36 |
| Cause of death |  |  | 0.11 |
| Anoxia | 7 (53.8%) | 3 (15.8%) |  |
| Cerebrovascular/  stroke | 5 (38.5%) | 10 (52.6%) |  |
| CNS Tumor | 0 (0%) | 1 (5.3%) |  |
| Head trauma | 1 (7.7%) | 5 (26.3%) |  |
| Cigarette use (>20 pack-years) | 0 (0%) | 3 (15.8%) | 0.29 |
| **Recipient** |  |  |  |
| Race |  |  | 0.75 |
| Black | 1 (7.7%) | 2 (10.5%) |  |
| White | 11 (84.6%) | 14 (73.7%) |  |
| Other | 1 (7.7%) | 3 (15.8%) |  |
| Gender (female) | 13 (100%) | 19 (100%) | >0.9 |
| Age | 57 (49-60) | 42 (38.2-55) | 0.02 |
| BMI | 25.4 (22.5-28.7) | 23.2 (20.2-27.7) | 0.36 |
| Serum creatinine (mg/dL) | 0.8 (0.7-0.8) | 0.8 (0.6-0.8) | 0.39 |
| Total bilirubin (mg/dL) | 0.4 (0.4-0.6) | 0.4 (0.3-0.5) | 0.30 |
| Time on waitlist (years) | 0.2 (0.1-0.6) | 0.5 (0.3-1.1) | 0.17 |
| Transplant type |  |  | 0.62 |
| Bilateral | 12 (92.3%) | 15 (78.9%) |  |
| Single | 1 (7.7%) | 4 (21.1%) |  |
| Ischemic time (hours) | 5.3 (4.5-5.7) | 5 (4.1-6.3) | 0.69 |

CNS=central nervous system, BMI=body mass index. Continuous variables reported as median (interquartile range), and categorical variables reported as n (%). Recipients with both pre- and post-transplant sirolimus use are included in the pre-transplant sirolimus group.

**Supplementary Table 4. Postoperative outcomes of patients undergoing lung transplant for lymphangioleiomyomatosis (LAM) with pre- vs. post-transplant sirolimus use.**

| **Variable** | **Pre-transplant sirolimus (n=13)** | **Post-transplant only sirolimus (n=19)** | **P** |
| --- | --- | --- | --- |
| Median survival (years) | 3.7 (lower 95% CI 1.9) | Lower 95% CI 11.8 (median not reached) | 0.003 (log-rank) |
| Length of stay (days) | 22 (16-31) | 17 (11-24.5) | 0.13 |
| Acute rejection | 1 (7.7%) | 0 (0%) | 0.36 |
| Airway dehiscence | 0 (0%) | 1 (5.3%) | >0.9 |
| Dialysis | 1 (7.7%) | 0 (0%) | 0.36 |
| Reintubation | 2 (15.4%) | 3 (15.8%) | >0.9 |

Survival reported as median (95% confidence interval), continuous variables reported as median (interquartile range), and categorical variables reported as n (%). Recipients with both pre- and post-transplant sirolimus use are included in the pre-transplant sirolimus group.

**Supplementary Figure 1. Post-lung transplant survival and 95% confidence interval for recipients with lymphangioleiomyomatosis (LAM) by age group, 2005-2021.**

**
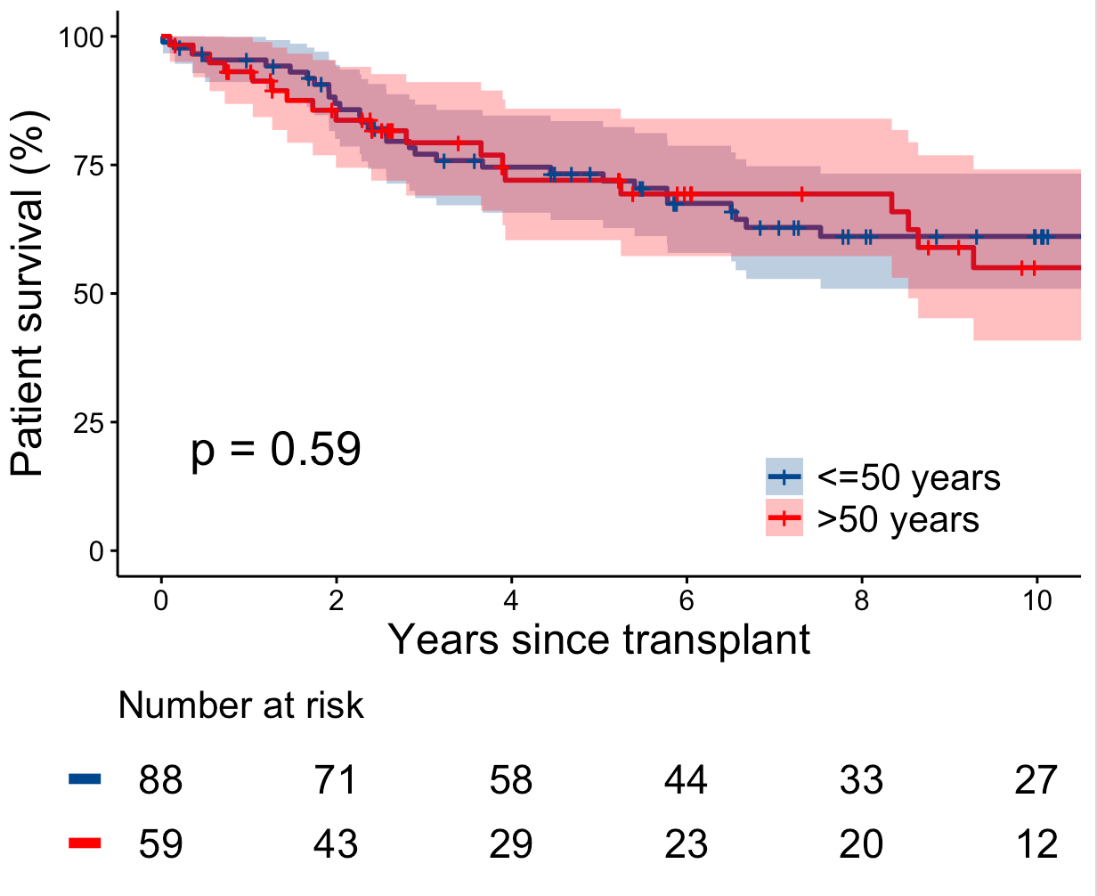
**
